# Techno-economic feasibility study of solar photovoltaic power plant using RETScreen to achieve Indonesia energy transition

**DOI:** 10.1016/j.heliyon.2024.e27680

**Published:** 2024-03-27

**Authors:** Hendry Timotiyas Paradongan, Dzikri Firmansyah Hakam, Sudarso Kaderi Wiryono, Iswan Prahastono, Indra A. Aditya, Kevin M. Banjarnahor, Ngapuli Irmea Sinisuka, Ayodele Asekomeh

**Affiliations:** a School of Business and Management, Bandung Institute of Technology, Bandung, 40132, Indonesia; b PLN Research Institute, PT PLN Persero, Jakarta, 12760, Indonesia; c School of Electrical Engineering and Informatics, Bandung Institute of Technology, Bandung, 40132, Indonesia; d Aberdeen Business School, Robert Gordon University, Aberdeen, AB10 1FR, Scotland, UK

**Keywords:** Renewable energy, Renewable energy tariffs, Energy transition, Photovoltaic power plant, Feasibility study, RETScreen, De-dieselization

## Abstract

Indonesia, a key player in the global energy transition, faces surging electricity demand and ambitious renewable energy goals. In response, the government introduced a new regulation about renewable energy tariffs, including tariffs for photovoltaic (PV). However, there remains a gap in the academic literature regarding PV power plant feasibility studies under these tariffs. To address this gap, this study investigates the feasibility of a utility-scale solar photovoltaic (PV) power plant in Indonesia, focusing on the newly implemented renewable energy tariffs based on Independent Power Producers (IPPs) and Indonesia's state-owned electricity company (PLN) perspectives. Five scenarios were developed based on the proposed 26 MW solar power plant on Nias Island utilizing RETScreen software. The results showed that based on the IPP perspective, the newly implemented renewable energy tariff was inadequate to make the project feasible, however, an introduction of a 10 USD/t CO_2_ emission incentive would make the project financially viable for IPPs. Therefore, it is recommended to introduce emission incentives as a strategic approach to attract investors and stimulate investment in Indonesia's PV power plants market, to accelerate Indonesia's energy transition. Conversely, the results also showed that the project is very profitable for PLN due to the significant cost-savings from the de-dieselization, leading to a reduction in the average generation cost for Nias.

## 1 Introduction

The world is highly dependent on electricity, with a projected increase in global electricity demand by 5900–7000 TeraWatt-hours (TWh) by 2030. This surge is equivalent to combining the current demand levels in the United States and the European Union [1]. Energy holds a critical role in Indonesia's economy, and the sustainable and equitable development of the energy sector is vital for the country's growth [2]. Indonesia has witnessed a 75% increase in electricity consumption from 2010 to 2020, averaging around 7.5% annually [3]. To overcome the energy demand with sustainable development, Indonesia has set a target of achieving a 23% renewable energy mix by the end of 2025 [[4], [5], [6]], and aims for net-zero emission in 2060 [7,8]. However, the current realization of Indonesia's renewable energy mix stands at only 11.5% by the end of 2021 [9]. Recognizing the urgency, the Indonesian government has introduced a new regulation for renewable energy tariffs [10], including for photovoltaic (PV) power plants.

Solar power plants are essential to human beings [11], not only for the potential to supply electricity but also for their help to mitigate CO_2_ emissions [[12], [13], [14], [15]]. Countries near the equator, benefitting from high solar irradiance, have witnessed rapid expansion in various types of solar power plants [[16], [17], [18]]. According to the Indonesian Ministry of Energy and Mineral Resources (ESDM), there are 112,000 GWp of solar energy potential in Indonesia [19], surpassing the country's existing installed capacity [20]. According to Kanugrahan's 2050 net-zero carbon scenario, PV power plants are expected to contribute to 56.95% of the total electricity generation output in Indonesia [21]. Based on those considerations, it is expected that solar energy will play a key part in achieving Indonesia's energy transition. Perusahaan Listrik Negara (PLN), the state-owned company responsible for electricity supply in Indonesia, has committed to lead the country's transition to renewable energy sources [22]. Unfortunately, PLN's solar power plant utilization in 2022 is only around 21.34 MW [23], representing a mere 0.00048% of PLN's total installed power capacity. One of the strategies being pursued by PLN is the de-dieselization program, which involves converting diesel-based power plants to renewable energy sources. A notable example is the plan to construct a 26 MW solar power plant in Nias, with a commercial operations date (COD) of 2025–2029.

The potential for solar energy to reduce electricity cost is substantial, Kassem et al. [24] evaluated the solar energy analysis and feasibility study of a 100 MW solar PV power plant in Northern Cyprus, the results showed an LCOE of 0.093 USD/kWh could be achieved, avoiding the emission of 2,906,917 tCO_2_ annually. In a study conducted by Kelly et al. [25] on off-grid PV, diesel, wind, and battery energy system options for isolated regions in Chad, the LCOE was found in the range of 0.367 USD – 0.529 USD, showing that in some sites, the LCOE is less than the average generation cost of Chad with 0.400 USD. Furthermore, Rehman et al. [26] conducted a feasibility analysis of a 10 MW PV power plant in Saudi Arabia with LCOE ranging from 0.183 USD/kWh to 0.049 USD/kWh. An LCOE study in Indonesia conducted by the Institute for Essential Services Reform (IESR) estimated the LCOE of PV power plants in Indonesia ranging from 0.103 USD/kWh to 0.058 USD/kWh [27].

Several studies have been conducted to estimate the LCOE of utility-scale PV power plants in different locations. A study by Lazard [28], a financial advisory and asset management firm, estimated the LCOE of utility-scale PV power plants in the United States in 2020 to be between 3.0 and 4.5 cents per kWh, depending on the location. The study found that the LCOE of PV power plants had decreased by 70% since 2009, making it competitive with conventional fossil fuel-based power plants in many regions. Another study by the National Renewable Energy Laboratory (NREL) [29] estimated the LCOE of utility-scale PV power plants in the United States to be between 3.8 and 7.2 cents per kWh, depending on the location and the type of technology used. The study found that the LCOE of PV power plants had decreased by 50% since 2010, making it one of the most cost-effective sources of electricity in many regions.

Several studies have investigated PV power plant feasibility studies outside of Indonesia. Pan et al. [30] conducted a feasibility analysis of various renewable technologies in Chongming, including various solar tracking modes, electricity tariffs, cost-savings, and emissions reduction incentives in the financial calculation which resulted in positive NPV. Other studies have also investigated about the feasibility of utility-scale photovoltaic power plants using RETScreen. For instance, studies [24,26,[30], [31], [32], [33], [34], [35], [36], [37]] examined utility-scale solar photovoltaic power plants outside Indonesia. Several studies have also examined off-grid solar photovoltaic power plants outside of Indonesia [[38], [39], [40], [41], [42], [43], [44], [45], [46], [47]]. Fathoni et al. [48] explored the feasibility of utility-scale solar photovoltaics in Indonesia using the feed-in tariffs in the financial calculation.

While several studies have explored the feasibility of utility-scale PV power plants, a crucial gap exists concerning Indonesia's newly implemented renewable energy tariffs. Furthermore, despite the efforts by the government to introduce new tariffs for PV power plants, Indonesia's PV power plant utilization is still lacking. Therefore, this research aims to address this gap by providing insights regarding the adequacy of the newly implemented tariffs for PV power plant project. It seeks to provide an understanding and solutions regarding Indonesia's solar energy issues, particularly from the perspectives of IPPs and PLN. In addition, to the best of author's knowledge, no prior research has been conducted to study the feasibility of the Nias 26 MW PV project. Therefore, this research serves as a pioneering effort to provide insights concerning the feasibility of the Nias 26 MW PV project based on the newly implemented renewable energy tariffs. The findings hopefully helped all stakeholders in the decision-making process and contribute to the realization of Indonesia's energy transition.

## 2 Literature review and Indonesia power system overview

### 2.1 Previous research

Studies on utility-scale photovoltaic (PV) power plants using RETScreen have been carried out in numerous locations, including those by Refs. [24,26,[30], [31], [32], [33], [34], [35], [36], [37], [38],[41], [42], [43], [44], [45], [46],[49], [50], [51]] and others have all used the RETScreen to evaluate the feasibility of small-scale photovoltaic power plants which proved RETScreen software is very capable to conduct not only the feasibility study of small-scale photovoltaic power plants but also utility-scale photovoltaic power plants.

The technical and financial potential of solar PV power plants with a feed-in tariff in various Indonesian locations was examined in 2014 by Fathoni et al. [48]. According to Fathoni's research, PV power plants are financially feasible in Indonesia, with payback periods ranging from 11 to 17.6 years. Notably, the city of Makassar emerged as the most financially viable location for solar PV power facilities in the country. However, it is important to mention that this study differs from Fathoni's study in terms of the calculation employed. Specifically, this study incorporates the cost savings of PLN in one of the scenarios. By considering PLN's cost savings, this study aims to provide a comprehensive and accurate assessment of the feasibility of PV power plant in Indonesia. Furthermore, this research distinguishes itself from previous studies, by incorporating the most updated PV tariffs specific to Indonesia, which to the best of author's knowledge, no previous study has been undertaken previously. By incorporating the most updated information on Indonesia's PV tariffs, this study fills a significant gap in the existing research. It offers a novel perspective on the feasibility of PV power plants in Indonesia by ensuring that the calculations and assumptions align with the current tariff regulations and current conditions.

All of the studies mentioned above look into a range of PV power plant-related topics, such as operational effectiveness, cost of energy production, power optimization, technological utilization, and economic viability. They shed light on issues including the evaluation of solar resources, the sizing of PV systems, financial feasibility, and regulatory frameworks, and they offer helpful insights into the design, installation, and optimization of PV power plants. Collectively, these publications add to the body of knowledge on utility-scale PV power plants and provide helpful resources for this research, in terms of valuable references and the global knowledge of PV power plants A comprehensive overview of the aforementioned earlier research, including their findings, initial cost costs, operation and maintenance costs, capacity, locations, power capacity, and financial incentives for renewable energy is shown in Table 1.

**Table 1:** Previous research on solar PV using RETScreen.

Author	Year	Electricity Rates (USD)	Renewable Incentives	Region	Capacity	Initial Cost (USD)	O&M Cost (USD/annum)	Results
[38]	2012	0.175/kWh	30 USD/tCO_2_	Iran	8 kW	110,000	1700	GHG incentives make the project more feasible with 6 years equity payback period and IRR of 21.9%.
[39]	2017	0.080/kWh	0.27 USD/tCO_2_	Dhahran, Saudi Arabia	12 kW	110,000	2134	GHG incentives make the project more feasible with 8.2 years equity payback period
[40]	2071	0.012–0.089/kWh	50% of initial cost	Iran	1 kW,3 kW,5 kW	3000/kW	Not specified	In the best scenario that consists of government incentives and a higher price of electricity, the IRR-asset is 33.3%.
[50]	2020	0.190/kWh	No grants or incentives	Northern Cyprus	3 kW	2800/kW	24.68/kW	The LCOE is projected to be lower than the current price of energy supplied from the grid.
[30] a	2017	0.096/kWh	5.71 USD/tCO_2_	Chongming, China	200 MW	78,104,714 (fixed-tilt) 96,961,857 (single-axis) 115,819,000 (double-axis)	100,000	Comparing PV tracking modes in PV systems, single-axis tracking system shows a significant 0.265 TWh increase in power generation. Despite longer payback periods and lower IRR than fixed tilt system, the single-axis brings the highest NPV, making it the most optimum
[41] b	2020	0.398/kWh	No grants or incentives	Bangladesh	4.7 kW	26,072	1996	Due to the lack of regional power and the availability of local resources, the economical solution involves the integration of PV panel, biogas generator plus battery storage.
[26]	2017	Not specified	No grants or incentives	Saudi Arabia	10 MW	13,440,108	500,000	The government should provide a subsidy and clean energy development incentives of 30–70% for attractive payback periods of 12 to 5 years.
[31] c	2020	0.012/kWh	No grants or incentives	Bati, Ethiopia	100 MW	5,785,714	Not specified	Feasible due to positive NPV result and lower energy production cost.
[42]	2011	Not specified	No grants or incentives	Australia	0, 20, 40. 60, 80, 100, 200 kW	2750/kW	Included in the initial cost	It is found that this type of energy system is feasible to solve the problem of rural electricity supply in Australia
[43] d	2014	Not specified	No grants or incentives	Pakistan	5 kW	2105	Not specified	Solar tracking system provide economic benefits in terms of reducing the number of PV panels required to fulfil energy needs
[32] e	2010	0.179/kWh	No grants or incentives	Northern Bangladesh	1 MW	3,887,759	14,143	The LCOE of the PV system is relatively lower than grid-connected diesel-based power cost.
[51]	2018	0.123/kWh	No grants or incentives	Surabaya, Indonesia	2070 kW	2070–5140	Not specified	The proposed PV system can provide annual electricity production of 3180 MWh or around 80% of the university energy demand.
[49]	2020	Not specified	No grants or incentives	Libya	10 MW	Not specified	Not specified	LCOE of PV power systems in selected regions of Libya ranging from 0.06 USD/kWh - 0.08 USD/kWh
[33]	2013	0.120/kWh	No grants or incentives	Quetta, Pakistan	10 MW	53,680,000	500,000	One-axis tracking system PV power plant generates the cheapest electricity compared to fixed-tilt and two-axis.
[44] f	2016	0.184/kWh (commercial) 0.071/kWh (residential)	0.06/kWh	Xi'an, China	3372 m^2^ (commercial) 7.45 m^2^ (residential)	308,548 (commercial) 1238 (residential)	116	The use of building-added PV could lower the cost of electricity by 46% with the use of hybrid energy system technology.
[45] g	2010	0.119/kWh	No grants or incentives	Northern Bangladesh	499.98 kW	1,720,238	7071	PV-based electricity production cost is lower than the grid-connected diesel power generation.
[34]	2019	0.12/kWh	10 USD/tCO_2_	Northeast Nigeria	6 MW	14,400,000	300,000	RETScreen software is accurate in calculating the total energy produced, the amount of GHG revenue, and the financial aspect.
[52]	2015	0.10/kWh	0.25 USD/kWh Feed-in tariff	Surabaya, Indonesia	1 kW	2800	Not specified	The feed-in tariff makes the project feasible with 6.5 years payback period compared to 17.6 years with regular tariff.
[48]	2014	0.25/kWh	Feed-in tariff included in electricity rates.	Indonesia	0.29–435.06 MW	4600/kW (>100 kW) 5300/kW (<100 kW)	50,000	Utilizing the feed-in tariff, solar power plant is financially feasible in Indonesia, with payback period ranging from 11 years to 17.6 years.
[35] h	2016	Not included	Not included	Germany	76.05 GW	1368/kW 1895/kW	36.9/kW	By 2030, photovoltaic systems projected to be cost-competitive with fossil fuels, with LCOE below 0.11 €/kWh. Roof-mounted systems having the highest cost reduction potential.
[36]	2020	0.12/kWh	10 USD/tCO_2_	Malaysia	5000 kW	6,800,000	65,000	The developed 7E approach can be applied to different energy systems at different locations and operating conditions.
[37]	2009	0.42/kWh	Not specified	Egypt	10 MW	103,740,822	334,500	Wahat Kharga 10 MW Connected Power Plant delivers the most cost-effective, power generation and GHG emission reductions

a Converted from CNY to USD – 1 USD = 7 CNY.

b Converted from BDT to USD – 1 USD = 84 BDT.

c Converted from ETB to USD – 1 USD = 28 ETB

d Converted from PKR to USD – 1 USD = 285 PKR

e Converted from BDT to USD – 1 USD = 84 BDT.

f Converted from CNY to USD – 1 USD = 7 CNY.

g Converted from BDT to USD – 1 USD = 84 BDT.

h Converted from EUR to USD – 1 USD = 0.95 EUR.

### 2.2 Indonesia's power system and renewable energy tariffs

**Fig. 1:**
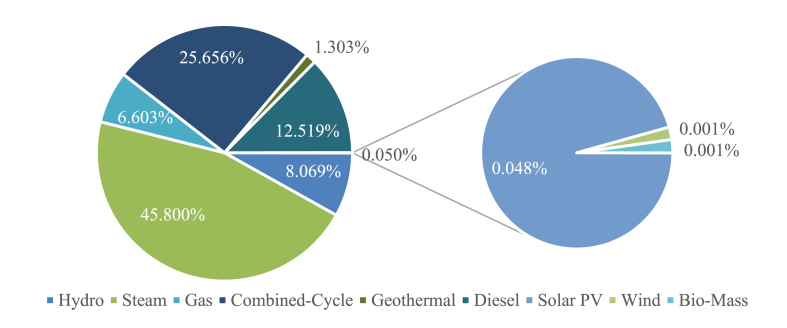
PLN's installed power capacity [23].

Indonesia is an archipelago nation with more than 17,500 islands and currently is the 4th most populous nation and the world's 16th largest GDP [53]. Indonesia's power system is made up of a mix of different types of power generation, including coal, diesel, natural gas, and renewable sources such as solar, wind, hydro, geothermal, and biomass. By 2021, according to Indonesia's Ministry of Energy and Mineral Resources [54], Indonesia's power plant capacity stands at 73,736 MW with coal-based power plants accounting for 65.93% of the total capacity. PLN and its subsidiaries operate 6143 power plants and generate 44,467.75 MW of electricity [23]. The overview of PLN's installed power capacity categorized based on the type of technology used is shown in Fig. 1.

**Fig. 2:**
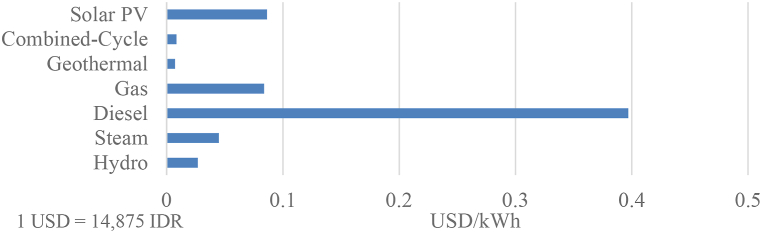
PLN's average electricity generation cost [23].

PLN selling tariffs are regulated by the government to ensure the affordability and availability of electricity [55], thus reducing the production cost of electricity becomes a crucial factor in increasing PLN's profitability. According to the most recent PLN's statistics report, PLN's average cost of electricity is 1333 IDR/kWh [23] or around 0.09 USD/kWh. However, it is worth mentioning that the cost of electricity from diesel power plants is significantly higher compared to other technologies, as illustrated in Fig. 2. Furthermore, PLN's selling prices in North Sumatera region is 1061 IDR/kWh [23], or approximately 0.07 USD/kWh. Considering these circumstances, it becomes evident that the cost of electricity from diesel power plants far exceeds PLN's selling prices to consumers, indicating that PLN incurs losses for every unit of electricity produced by its diesel power plants. To address this issue, PLN implemented a de-dieselization program, which aims to transitioning diesel power plants into renewable energy power plants.

**Fig. 3:**
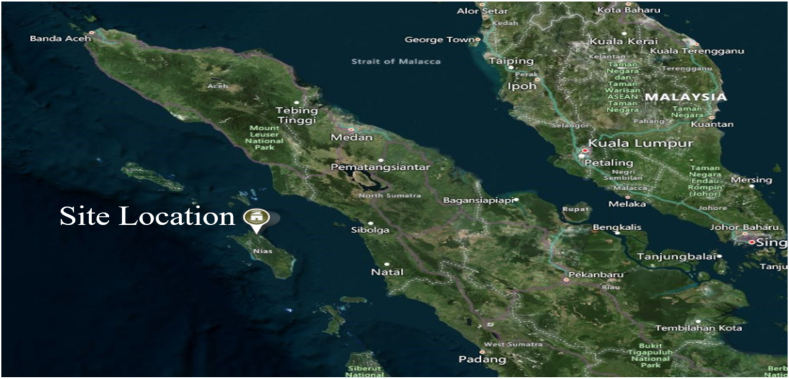
Site location.

Recently, the Indonesian government released a regulation regarding the acceleration of renewable energy development [10], which includes the regulation of tariffs for renewable energy sources in Indonesia, including solar PV power plants. Several considerations were included in the tariffs, including the technology type, power plant location, and power plant capacity. The regulation further classified the tariffs into two categories, expansion power plants, and initial power plants. Furthermore, the renewable energy tariffs were divided into two timeframes, years 1–10 and years 11–30. The proposed site for the power plant is planned to be located in the northern part of Nias, North Sumatera, as illustrated in Fig. 3.

As shown in Table 2 and Table 3, there is an inverse correlation between capacity and tariff rates, indicating an approach to incentivize and promote decentralized solar energy generation. The tariff structure encourages smaller-scale projects to benefit from higher tariff rates to participate in the renewable energy sector. On the other hand, the tariff structure for larger-scale projects is lower compared to smaller-scale projects. The tariffs will be converted from USD to IDR using the Central Bank Indonesia (BI) Jakarta Interbank Spot Dollar Rate (JISDOR) [56]. Currency rates that will be used in this study is the average currency in 2022 or equal to Rp. 14,875.00.

**Table 2:** PV power plant tariffs for Nias region [10].

**Capacity**	**Tariff Year 1**–**10**	**Tariff Year 11**–**30**
**≤1 MW**	0.1319 USD	0.0688 USD
**>1 MW-3 MW**	0.1143 USD	0.0597 USD
**>3 MW-5 MW**	0.1009 USD	0.0526 USD
**>5 MW-10 MW**	0.0950 USD	0.0496 USD
**>10 MW–20 MW**	0.0913 USD	0.0476 USD
**>20 MW**	0.0799 USD	0.0417 USD

**Table 3:** PV power plant expansion tariffs for Nias region [10].

**Capacity**	**Tariff Year 1**–**10**	**Tariff Year 11**–**30**
**≤1 MW**	0.1055 USD	0.0550 USD
**>1 MW-3 MW**	0.0914 USD	0.0478 USD
**>3 MW-5 MW**	0.0807 USD	0.0421 USD
**>5 MW-10 MW**	0.0760 USD	0.0397 USD
**>10 MW–20 MW**	0.0730 USD	0.0381 USD
**>20 MW**	0.0639 USD	0.0334 USD

### 2.3 De-dieselization program: Nias case

In order to boost Indonesia's renewable energy mix to achieve the 23% target by 2025, PLN launched the diesel conversion to renewable energy program, also known as the De-dieselization program [57]. This program aims to reduce the cost of electricity generation, decrease CO_2_ emission, and increase the mix of renewable energy [22]. As part of the De-dieselization program, PLN is planning to convert several diesel power plants, located on Nias Island into 26 MW PV power plant.

**Fig. 4:**
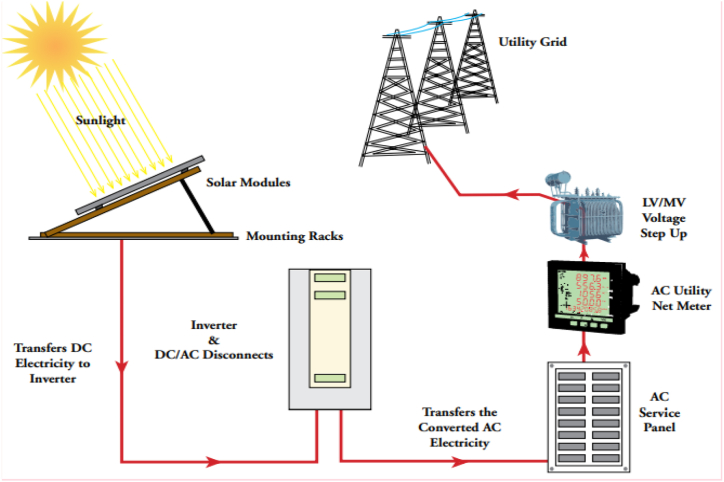
Configuration of utility-scale PV power plant [58].

As shown in Table 4, Nias power system is mainly powered by diesel, which tends to have higher electricity production cost compared to others. Several PLN owned power plants are Gunung Sitoli Machine Gas Fired Power Plant and Gunung Sitoli Diesel Power Plant with 41.1 MW installed capacity. Lease and IPP power plants contribute to 46.8 MW of Nias power system. As part of PLN's de-dieselization program, several diesel power plants will be converted to 26 MW solar PV power plant in, aiming to increase Indonesia's renewable energy mix and decrease PLN's production cost. An overview of PV power plant configuration is shown in Fig. 4.

**Table 4:** Power plants in Nias power system.

Power Plant Name	Fuel Type	Ownership	Installed Capacity	Production Capacity
Nias 25 MW Ga Mobile Power Plant	Gas	IPP	27 MW	25 MW
Gunung Sitoli Machine Gas Fired Power Plant	Gas Diesel	PLN	34.5 MW	30 MW
Gunung Sitoli Diesel Power Plant	Diesel	PLN	6.6 MW	6 MW
Gunung Sitoli Lease Diesel Power Plant	Diesel	Lease	8 MW	6.4 MW
Teluk Dalam Diesel Power Plant	Diesel	Lease	11.8 MW	4.2 MW
Total Capacity			87.9 MW	71.2 MW
Total Peak Load in Nias System			37.0 MW	37.0 MW
Capacity Reserved			50.9 MW	34.2 MW

## 3 Research methodology and data collection

### 3.1 Research framework

**Fig. 5:**
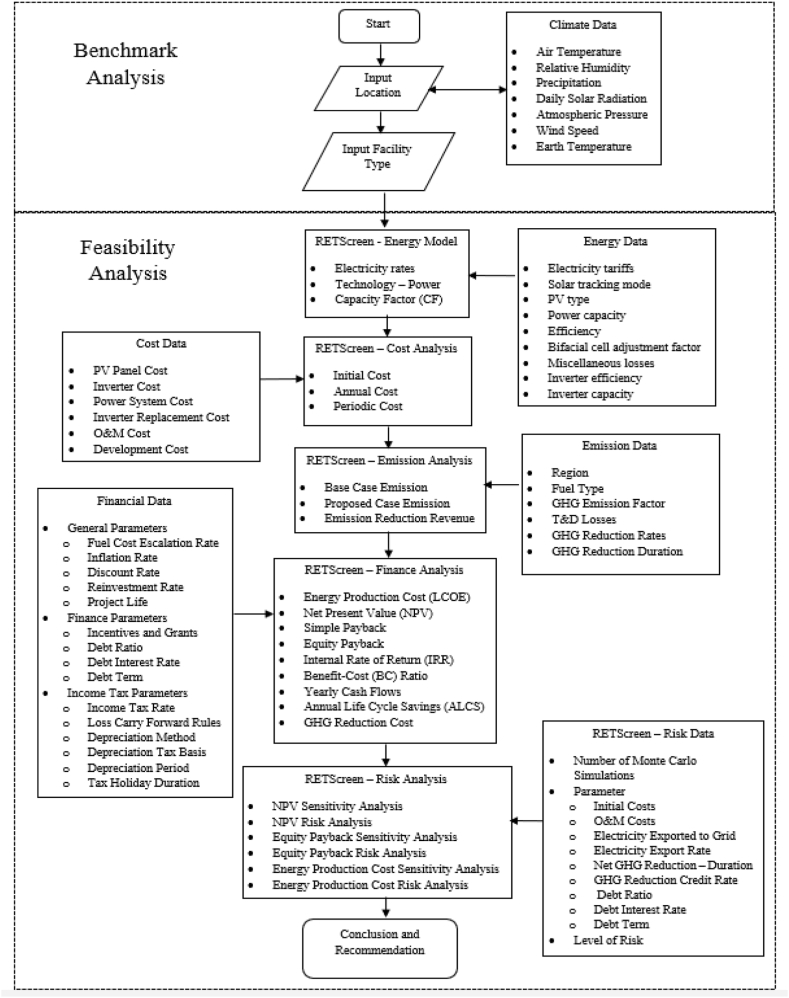
Research methodology.

RETScreen is a software capable to conduct feasibility analysis for various renewable energy based on the scenario inputted by the user. Scenario is the condition of the projected energy system that evolves in the specific constraint included in RETScreen worksheets. These worksheets included climate data, energy data, cost data, emission data, financial data, and risk data. The overview of the feasibility analysis process for the Nias 26 MW PV power plant is shown in Fig. 5.

### 3.2 Tariffs scenarios

The new renewable energy tariff framework incorporates crucial factors like power plant capacity, geographical location, and operational duration. Specifically, the PV tariffs [10] distinguish between initial and expansion capacities. For instance, a 5 MW initial construction with an additional 5 MW in subsequent years incurs a different tariff than a single 10 MW initial capacity. To assess the adequacy of these tariffs for PV power plant projects, five distinct scenarios were developed, considering various perspectives. The base case scenario represents the perspective of Independent Power Producers (IPPs) by employing the 26 MW PV tariff. RUPTL scenario represents the IPPs perspective with an initial 6 MW capacity and a subsequent 20 MW expansion. The proposed case scenario, suggested by the authors, involves a 10 MW initial capacity and a 16 MW expansion, utilizing 10 MW initial capacity and 16 MW expansion tariffs. The cost-savings scenario integrates the perspective of PLN, market, with 10 USD/tCO_2_ emission incentives for every green-house gas (GHG) emission reduction. The overview of each scenario tariff is presented in Table 5.

**Table 5:** Tariffs scenario.

Scenario	Initial capacity (MW)	**Expansion capacity (MW)**	Average tariffs (USD/kWh)	Cost savings (USD/kWh)	Incentives (USD/tCO_2_)
**Base case**	26	0	0.0570	n/a	n/a
**RUPTL**	6	20	0.0573	n/a	n/a
**Proposed case**	10	16	0.0594	n/a	n/a
**Cost-savings**	10	16	0.0594	0.0572	n/a
**Clean energy**	10	16	0.0594	n/a	10

### 3.3 Climate data

The 26 MW solar PV power plant location was planned to be located in Northern Nias, North Sumatra. The variations of climate data used in the energy modelling, including air temperature, relative humidity, precipitation, daily solar radiation, atmosphere pressure, wind speed, and earth temperature are shown in Table 6.

**Table 6:** Site Climate Data [59].

Month	Air Temperature (°C)	Relative Humidity (%)	Precipitation (mm)	Daily solar radiation (kWh/m^2^/d)	Atmosphere pressure (kPa)	Wind speed (m/s)	Earth temperature (°C)
January	27.4	79.1	209.25	5.05	100.69	2.83	28.9
February	27.7	78.0	179.76	5.27	100.69	2.86	29.3
March	27.8	79.2	246.14	5.07	100.67	3.07	29.3
April	27.8	80.1	236.10	4.77	100.63	2.94	29.3
May	28.2	78.3	197.78	4.95	100.61	2.77	29.5
June	28.0	77.3	163.50	4.83	100.65	2.92	29.4
July	27.7	77.6	214.21	4.61	100.69	3.13	29.2
August	27.5	78.6	239.32	4.60	100.71	3.13	29.1
September	27.3	79.8	270.00	4.58	100.73	3.28	28.8
October	27.1	81.2	315.27	4.48	100.72	3.50	28.5
November	27.0	82.3	345.60	4.32	100.68	3.53	28.4
December	27.1	80.9	284.27	4.68	100.70	2.99	28.5
Annual	27.5	79.4	2901.20	4.76	100.68	3.08	29.0

### 3.4 Energy and emission modelling

The energy production calculation in this research was conducted using RETScreen. The Capacity Factor (CF), signifying the amount of electricity generated by a power plant relative to its maximum capacity was determined using Eq. **(1)**. The PV panel specification data used in this study was shown in Table 7. The inverter specification data used in the calculation in this study was shown in Table 8.

(1) $$CF=\frac{Net\;generated\;electrical\;energy}{Nominal\;Capacity}\times 100$$

**Table 7:** PV panels specification.

Photovoltaic panel type	Mono-Crystalline
Solar tracking mode	Fixed
Capacity	575 Wp
Efficiency	21.3 %
Dimension	2.704 m^2^
Bifacial cell adjustment factor	30 %
Number of modules	46,000
Cost	225 USD/unit
Lifetime expectancy	25 years
Miscellaneous losses	5 %

**Table 8:** Inverter specification.

Capacity	26,000 kW
Efficiency	97%
Lifetime expectancy	25 years
Cost	250 USD/kW

The emission reduction calculation in this study was conducted using RETScreen software, which enables the modeler to estimate the GHG emission reduction of a potential project [60]. The GHG emission reduction was calculated using Eq. **(2)**, and the GHG emission reduction revenue was calculated using Eq. **(3)**.

(2) ΔGHG=ABCE−APCE

(3) GRR=ΔGHG×GHGemissionincentives

Where *ΔGHG* is the emission reduction, *ABCE* is the annual base case emission, *APCE* is the annual proposed case emission, GHG emission incentives are the amounts of incentives granted per ton CO_2_ reduction. The overview of the baseline data used in the calculation was shown in Table 9.

**Table 9:** Baseline electricity system emission data.

Region	Fuel type	GHG emission factor	**T&D losses (%)**
**Indonesia**	All types	0.755	9

### 3.5 Finance and risk modelling

The financial modelling in this study was conducted utilizing RETScreen software. The financial modelling started with cash flow projection, cash flow is essentially the difference between the cash inflow and the cash outflow calculated using Eq.**(4)**. Cash inflow was calculated using Eq. **(5)**, cash outflow was calculated using Eq. **(6)**.

(4) Cn=Cin,n−Cout,n

(5) Cin,n=Cener,n+CS,n+CGHG,n

(6) $$Cout,n=\left(CO\&M\right){\left(1+ri\right)}^{n}+id,n\frac{\left(debt\right)}{{1-\left(1+id,n\right)}^{-N^{\prime }}}$$

Where *Cn* is the cash flow for year *n*, *Cin,n* is the cash inflow for year *n*, *Cout,n* is the cash outflow for year *n*, *Cener,n* is the energy income for year *n*, *CS,n* is income resulting from cost savings for year *n*, *CGHG,n* is the GHG reduction income for year *n*, *CO&M* is operation and maintenance cost, *ri* is the inflation rate, *id,n* is the effective debt interest rate at year *n*, *debt* is the amount of debt, *N′* is the debt term.

The Net Present Value (NPV) which represents the profitability of an investment or a project was calculated using Eq. **(7)**. Levelized Cost of Electricity (LCOE), a metric used to assess the average cost of electricity generation from a power plant over its lifetime was calculated using Eq. **(8)**. Simple Payback (SP) representing the number of years of cash flow to equal the initial cost and does not consider the time value of money factor was calculated using Eq. **(9)**. The Equity Payback (EP) which represents the number of years needed to generate positive cash flow, was calculated using Eq. **(10)**. Internal Rate of Return (IRR), defined as the discount rate at which the NPV of the project becomes zero is calculated by solving Eq. **(11)**. Other important parameters such as Benefit-Cost Ratio (B–C), Annual Life Cycle Savings, GHG Reduction Cost (GRC), were calculated using Eq. **(12)**, Eq. **(13)**, Eq. **(14)**, respectively.

(7) $$NPV=\sum_{n=0}^{N}\frac{Cn}{{\left(1+r\right)}^{n}}-IC$$

(8) $$LCOE=\frac{sum\;of\;cost\;over\;lifetime}{sum\;of\;electricity\;generated\;over\;lifetime}$$

(9) $$SP=\frac{IC-IG}{\left(Cener+CS+GRR\right)-\left(CO\&M+Cfuel\right)}$$

(10) $$EP=\sum_{n=0}^{NPCF}Cn$$

(11) $$IRR=0=\sum_{n=0}^{N}\frac{Cn}{{\left(1+IRR\right)}^{n}}$$

(12) $$B-C=\frac{\sum_{n=0}^{N}\frac{Cn}{{\left(1+r\right)}^{n}}}{IC}$$

(13) $$ALCS=\frac{NPV}{\frac{1}{r}\;\left(1-\frac{1}{{\left(1+r\right)}^{N}}\right)}$$

(14) $$GRC=\frac{NPV}{\Delta GHG}$$

Where *N* is the project life in year, *r* is the discount rate, *IC* is the initial cost, *IG* is the incentive and grant, *Cfuel* is the cost of fuel, which is zero for this PV project, *PCF* is the positive cash flow, *B* is the benefit resulting from the project, *C* is the cost incurred by the project. The overview of financial input data used in this study is shown in Table 10.

**Table 10:** Financial input data.

Financial inputs	Value
**Initial costs**	25,975,000 USD
**Annual O&M costs**	13 USD/kW
**Discount rate**	7%
**Inflation rate**	5.71%
**Project lifetime**	25 years
**Debt ratio**	70%
**Debt term**	15 years
**Debt interest rate**	8%
**Electricity tariff escalation rate**	0%

Given the project's lifetime of 25 years, the inflation rate is considered by employing the average inflation rate for the last 25 years, calculated from BI inflation data [61]. The debt ratio is based on several pieces of literature [30,48,60], which used 70% as the debt ratio. The debt interest rate is based on corporate credit interest rate, obtained from Indonesia's Financial Services Authority (OJK) [62]. Electricity tariff escalation rate is not considered due to Indonesia's fixed-rate system. Other financial input data were gathered from RETScreen database, previous studies, PLN's reports [23,63], and other relevant literature.

This study employs Monte Carlo simulations to perform risk analysis, a highly effective technique for enhancing the precision of estimators regarding the performance of the model [64], conducted utilizing RETScreen software. The project's associated risk was assessed to gain an understanding of its implications. Multiple parameters, as outlined in Table 11, were employed as inputs for the risk analysis. Through the evaluation of the impact of these input parameters on the NPV, an estimation was made regarding the risk entailed by the project, probabilities of success, and the identification of the key parameter.

**Table 11:** Risk parameter.

Parameter	Unit	Value	Range (%)	Min.	Max.
Initial cost	USD	25,975,000	25	19,481,250	32,468,750
O&M cost	USD	338,000	25	253,500	422,500
Electricity output to grid	MWh	49,575.79	25	37,181.84	61,969.73
Electricity rate base case scenario	USD/MWh	56.99	25	42.74	71.24
Electricity rate RUPTL case scenario	USD/MWh	57.33	25	43.00	71.66
Electricity rate proposed case scenario	USD/MWh	59.41	25	44.57	74.26
Electricity rate cost-savings scenario	USD/MWh	59.41	25	44.57	74.26
Electricity rate clean energy scenario	USD/MWh	59.41	25	44.57	74.26
Cost savings	USD/MWh	57.23	25	42.92	71.54
Net GHG reduction	tCO_2_	955,764	25	716,823	1,194,705
GHG reduction rate	USD/tCO_2_	10	25	7.5	12.5
Debt ratio	%	70	25	52.5	87.5
Debt interest rate	%	8	25	6	10
Debt term	Year	15	25	11.25	18.75

## 4 Results and discussion

### 4.1 Energy and emission analysis

The solar photovoltaic power plant technical analysis results provide key parameters that offer insights into the performance and characteristics of the facility. The capacity factor is calculated at 21.8%, signifying 21.8% electricity generation is achieved relative to its maximum capacity, corresponding to 49,576 MWh annually. The levelized cost of electricity (LCOE) indicating the average cost of producing electricity over the plant's lifespan, is 0.0595 USD/kWh. The relatively higher LCOE in this study compared to Rehman's research [26] can be attributed to differences in solar irradiance, resulting to differences in capacity factors. Additionally, the absence of renewable energy incentives covering the initial cost of the power plant has also contributed to the difference in LCOE. However, the project LCOE, at 0.0595 USD/kWh is considerably lower than the average generation cost in Nias. The solar collector area specifies the cumulative surface area of the solar panels in the power plant, projected to be 124,359 m^2^. This measurement serves as an indicator of the extent of solar panel coverage, thereby influencing the overall required area of the plant. Nominal operating cell temperature signifying the average temperature at which the solar cells operate during normal functioning, is 45 °C (°C). The temperature coefficient indicates the relationship between temperature variations and solar cell efficiency. The coefficient of 0.4% per degree Celsius indicates that for every 1 °C increase in temperature, the efficiency of the solar cells decreases by 0.4%. A summary of the project's technical performance is presented in Table 12.

**Table 12:** Technical analysis results.

Parameter	Value
**Capacity factor (%)**	21.8
**Levelized cost of electricity (USD/kWh)**	0.0595
**Annual electricity exported to grid (MWh)**	49,576
Solar collector area (m^2^)	124,359
**Nominal operating cell temperature (°C)**	45
**Temperature coefficient (%/°C)**	0.4

**Fig. 6:**
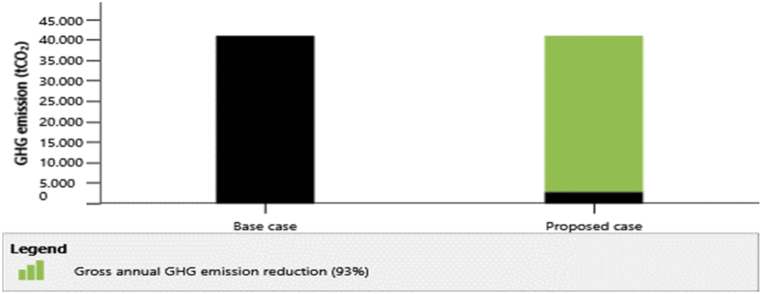
Annual GHG emission reduction.

In the emission analysis, the study's baseline scenario involves evaluating the emissions generated by Indonesia's power plants using the RETScreen database, with a corresponding GHG emission factor of 0.755 t CO_2_/MWh. Following the consideration of 9%, the GHG emission factor is calculated at 0.833 t CO_2_/MWh. The study's findings are depicted in Fig. 6, which demonstrates that the construction of a 26 MW solar power plant under all five scenarios led to a sizable reduction in gross yearly GHG emissions of 38,230.6 tCO_2_. To put into context, this is equivalent to avoiding the yearly usage of around 16,511,760.9 L of gasoline. If the clean energy scenario's emission reduction incentives are taken into account, the anticipated gross yearly GHG emission reduction of 38,230.6 tCO_2_ would result in an increase in revenue of 382,306 USD.

### 4.2 Financial analysis

The financial analysis of a project holds significant importance in gauging its financial viability. The financial analysis worksheet of RETScreen software enables the user to input economic parameters that include the rate of inflation, discount rate, debt ratio, debt interest rate, debt term, etc. RETScreen software calculated the cumulative cash flow, NPV, IRR, simple payback period, equity payback period, LCOE, etc.

**Fig. 7:**
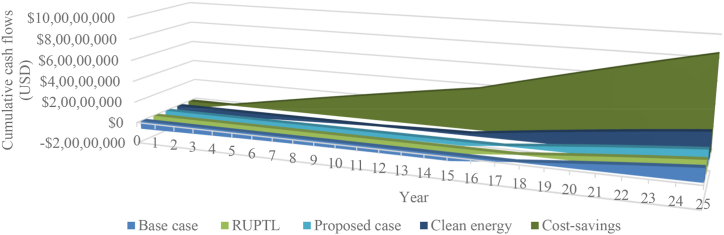
Cumulative cash flows of all scenarios.

Cumulative cash flows for all scenarios are shown in Fig. 7. As shown in the figure, the cumulative cash flows for five scenarios of the 26 MW solar PV power plant project are 12,155,922 USD, 12,579,922 USD, 15,155,257 USD, 24,712,897 USD, 86,086,199 USD for base case, RUPTL, proposed case, clean energy, and cost savings scenarios respectively. Based on those result, cost-savings scenario brings the highest cumulative cash flows, followed by clean energy, proposed case, RUPTL, and lastly base case scenario.

**Fig. 8:**
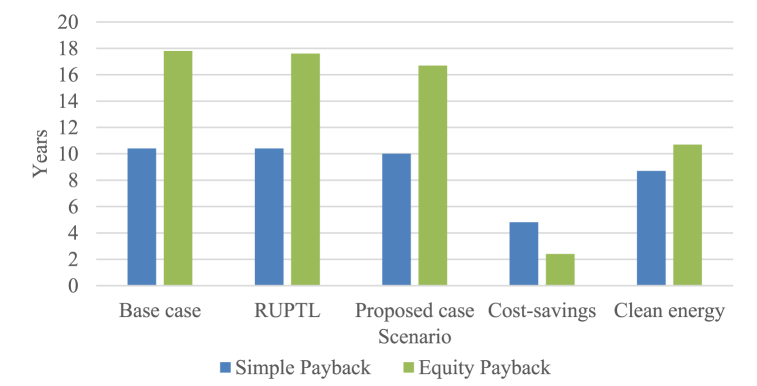
Payback periods of all scenarios.

Fig. 8 showed that in the base case and the RUPTL case, the simple payback period is 10.4 years, which signifies the time required to recover the initial investment. However, the proposed case exhibits a shorter simple payback period of 10 years, indicating a quicker return on investment. The cost-savings scenario demonstrates the shortest simple payback period of 4.8 years, while the clean energy case has a simple payback period of 8.7 years. It is important to mention, that the introduction of GHG reduction incentive has been effective in shortening the simple payback period by 13%.

The equity payback period denotes the time required to recover the investment solely from equity. In the base case, the equity payback period is 17.8 years, while the RUPTL case shows a slightly shorter period of 17.6 years. In contrast, the proposed case exhibits a further reduction in the equity payback period to 16.7 years. The cost-savings scenario has the shortest equity payback period of 2.4 years, indicating a rapid recovery of equity. The clean energy case displays an equity payback period of 10.7 years. The cost-savings equity payback and simple payback period are comparatively similar to Ref. [30], primarily attributed to the similar cost-savings amount per kWh. It is necessary to point out that, the introduction of GHG reduction incentive has been effective in shortening the equity payback period by 35.9%, signifying the effectiveness of GHG reduction incentive in expediting the payback period.

A summary of financial parameters and the corresponding values for each scenario is presented in Table 13. Despite the positive cash flow and a payback period less than the project's lifetime, the NPV results for the base case, RUPTL, and proposed case are negative, indicating the project tends to be unprofitable based on these scenarios. In the base case, the NPV is −1,459,834 USD, indicating a negative net value considering the time value of money factor. Similarly, the RUPTL case shows a negative NPV of −1,262,249 USD. However, the proposed case displays a relatively smaller negative NPV of −61,714 USD. Conversely, the cost-savings scenario exhibits a substantial positive NPV of 33,002,234 USD, indicating a high profitability due to significant cost savings. The cost-savings results is relatively close to Pan's research [30], particularly in terms of IRR. However, a notable disparity exists in the NPV, attributable to our utilization of 7% discount rate compared to Pan's 12% discount rate, with a lower discount rate generally yielding higher NPV due to augmented present value of future cash flows. The clean energy case also shows a positive NPV of 4,393,516 USD indicating that the GHG reduction incentives would make the project financially viable, therefore, it is suggested to introduce emission-incentives as a strategic approach to attract more investors, to accelerate the country's energy transition. This result align with [34,38,65] that also finds emission-incentives as an important factor to make PV project more financially feasible.

**Table 13:** Financial analysis results of all scenarios.

Parameter	Base case	RUPTL	Proposed case	Cost-savings	Clean energy
**Net present value (USD)**	−1,459,834	−1,262,249	−61,714	33,002,234	4,393,516
**Levelized cost of electricity (USD/kWh)**	0.0595	0.0595	0.0595	0.0595	0.0595
**Simple payback (Year)**	10.4	10.4	10	4.8	8.7
**Equity payback (Year)**	17.8	17.6	16.7	2.4	10.7
**Internal rate of return (%)**	5.6	5.8	6.9	41.8	11.3
**Benefit-cost ratio**	0.81	0.84	0.99	5.2	1.6
**Annual life cycle savings (USD/year)**	−125,269	−108,314	−5296	2,831,939	377,010
GHG emission reduction cost (USD/tCO_2_)	3.26	2.82	0.138	−73.69	−9.86

The base case exhibits an IRR of 5.6%, while the RUPTL case shows a slightly higher IRR of 5.8%. In the proposed case, the IRR further increases to 6.9%, indicating improved profitability. However, the IRR is lower than the discount rate used in this study, indicating that the rate of return of less than the desired outcome. The cost-savings scenario displays a significantly higher IRR of 41.8%, indicating substantial returns. The clean energy scenario demonstrates an IRR of 11.3%, a 4.4% IRR increase compared to the proposed case. The benefit-cost ratio compares the present value of benefits to the present value of costs. The base case and the RUPTL case have benefit-cost ratios of 0.81 and 0.84, respectively, indicating that the benefits are lower than the costs. However, the proposed case shows a benefit-cost ratio of 0.99, indicating nearly equal benefits and costs. The cost-savings scenario exhibits the highest benefit-cost ratio of 5.2, indicating substantial benefits outweighing the costs due to significant cost savings from the de-dieselization. The clean energy scenario has a benefit-cost ratio of 1.6, indicating a higher benefit as a result of GHG incentive.

The annual life cycle savings represent the net savings achieved each year over the project's lifetime. In the base case, the annual life cycle savings are −125,269 USD/year, indicating a net loss. The RUPTL case shows slightly improved savings of −108,314 USD/year. In the proposed case, the annual life cycle savings improve further to −5296 USD/year, indicating a smaller net loss. The cost-savings case exhibits significant annual savings of 2,831,939 USD/year, while the clean energy case displays annual savings of 377,010 USD/year. Lastly, the GHG emission reduction cost represents the cost of reducing one ton of greenhouse gas emissions. In the base case, the cost is 3.26 USD/t CO_2_, while the RUPTL case shows a slightly lower cost of 2.82 USD/t CO_2_. In the proposed case, the GHG emission reduction cost significantly decreases to 0.138 USD/t CO_2_, indicating a more cost-efficient result due to higher NPV compared to base case and RUPTL. The cost-savings case displays a negative GHG emission reduction cost of −73.69 USD/tCO_2_, implying that it is a cost-saving measure. The clean energy case also demonstrates a negative GHG emission reduction cost with −9.86 USD/t CO_2_.

### 4.3 Sensitivity analysis

**Fig. 9:**
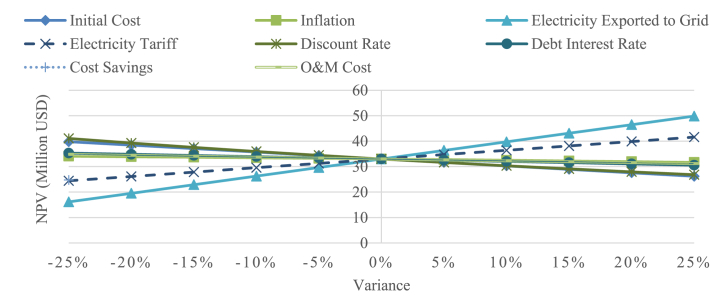
Sensitivity diagram of cost-savings scenario.

Sensitivity analysis underscores the varying significance of key parameters in determining the viability of the project, as measured by their impact on the net present value. Parameters examined include the initial cost, inflation rate, electricity exported to the grid, electricity tariff, discount rate, debt interest rate, cost savings, and O&M cost with base values of $25,975,000 USD, 5.71%, 49,576 MWh, $0.0594 USD/kWh, 7%, 8%, $57.23/MWh, and 13 USD/kW respectively. Employing a 25% range sensitivity analysis aimed at identifying the most critical parameters to the project's profitability. The results depicted in Fig. 9 reveal that electricity exported to the grid as the most influential parameter, due to its significant impact on cost-savings and revenue increase from electricity sales. The more electricity exported to grid, the more profitable the project is, vice versa. Following closely in importance is the electricity tariff, underscoring the significance of a fair tariff structure for investor appeal. Cost savings emerge as the third critical parameter, where higher cost savings correlate with increased project profitability. This underscores the strategic importance of selecting regions with substantial cost savings, particularly areas still reliant on diesel power plants. Notably, the NPV exhibits variances of 18.7% and 20.56.% for the discount rate and initial cost, respectively, when these parameters increase by 25%. Additionally, changes in O&M costs result in a 5.49% variation in NPV under a 25% parameter increase. Comparatively, the inflation rate appears to have the least significant impact, contributing to 5.49% NPV decrease when the parameter is increased by 25%.

Examining a comprehensive set of parameters, the study encompasses the initial cost, inflation rate, electricity exported to the grid, electricity tariff, discount rate, debt interest rate, GHG reduction credit rate, and O&M cost. These parameters hold base values of $25,975,000 USD, 5.71%, 49,576 MWh, $0.0594 USD/kWh, 7%, 8%, $10 USD/tCO2, and $13 USD/kW, respectively. Conducting a 25% range sensitivity analysis, the aim is to provide insights into the key factors influencing the project's profitability, with a specific focus on understanding the impact of GHG emission incentives if implemented in Indonesia.

**Fig. 10:**
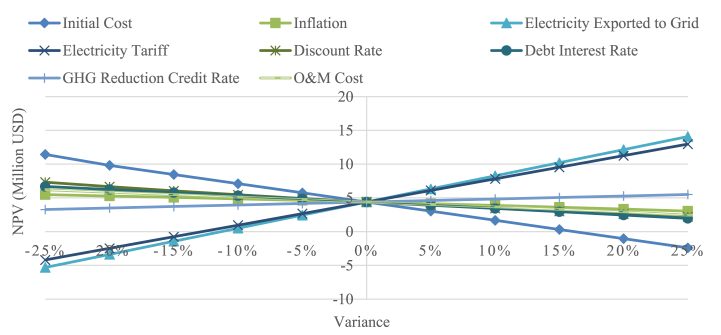
Sensitivity diagram of clean-energy scenario.

The results, as depicted in Fig. 10, shows electricity exported to the grid as the most influential factor, with 5% variation resulting in 44.13% NPV difference. This underscores the importance of selecting regions characterized by high solar irradiance levels to ensure the project's profitability. Closely following in importance is the electricity tariff, underscoring the pivotal role of a fair tariff structure for profitability of PV project. Initial Cost, while substantial, ranks third in impact, with a variance of approximately 61.7% variation in NPV under 10% parameter shift. Discount rate reflects the time value of money and influences the present value of future cash flows, making overall market condition as an important factor. For example, if there are attractive alternative investment opportunities with higher expected returns, investors may demand a higher discount rate, due to the opportunity cost they missed. Notably, the NPV exhibits variances of 10.81% and 8.25%, for debt interest rate and O&M cost, respectively. Additionally, inflation parameter contributes to 55,63% NPV difference, when this parameter varies by 25%. GHG credit rate emerges as the least influential factor, with 5.07% NPV difference by a 5% parameter shift. However, it is worth mentioning, that GHG credit played a pivotal role in making the project feasible, particularly if conducted by an IPP, evident in the positive NPV result it contributed.

**Fig. 11:**
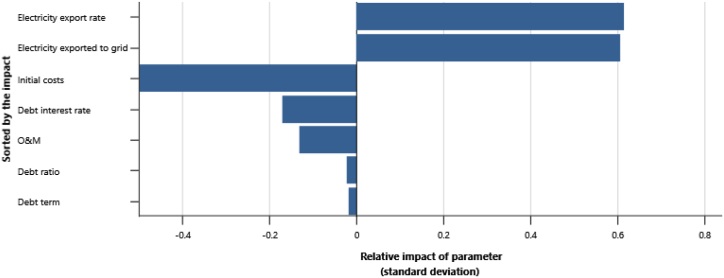
Base case sensitivity analysis – NPV.

**Fig. 12:**
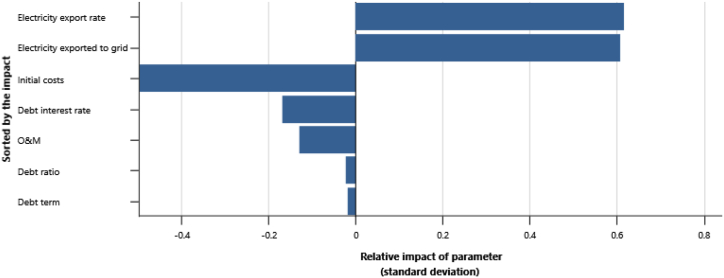
RUPTL sensitivity analysis – NPV.

**Fig. 13:**
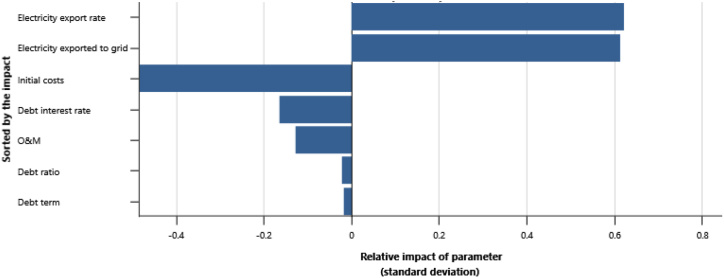
Proposed case sensitivity analysis – NPV.

**Fig. 14:**
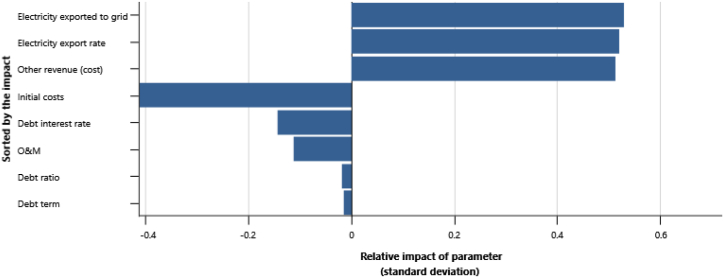
Cost savings sensitivity analysis – NPV.

**Fig. 15:**
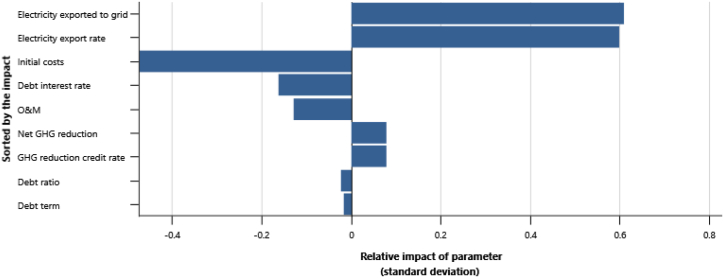
Clean energy sensitivity analysis – NPV.

The IPPs perspective which represented in base case, RUPTL, and proposed case sensitivity analysis were shown in Fig. 11, Fig. 12, Fig. 13. The most sensitive parameter to NPV based on the IPPs perspective is the electricity export rate, the higher the electricity export rates the more profitable the project is, and vice versa. The second most sensitive is electricity exported to grid, followed by initial costs, debt interest rate, O&M cost, debt ratio and lastly debt term. Fig. 14 represents PLN's point of view, the most sensitive parameter to NPV is the electricity exported to grid, due to its impact on both cost-savings and revenue from electricity sales. The second most sensitive is electricity export rate, cost savings, initial costs, debt interest rate, O&M cost, debt ratio and lastly debt term. Fig. 15 shows that the most sensitive parameter to NPV is the electricity export rates, the higher the electricity export rates the more profitable the project is, the lower the electricity export rates, the more unprofitable the project. The second most sensitive is electricity exported to grid, followed by initial costs, debt interest rate, O&M cost, net-GHG reduction, GHG reduction credit rate, debt ratio and lastly debt term.

### 4.4 Risk analysis

**Fig. 16:**
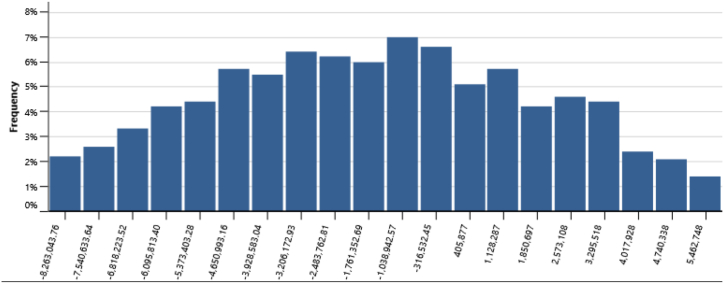
Distribution of base case scenario – NPV.

**Fig. 17:**
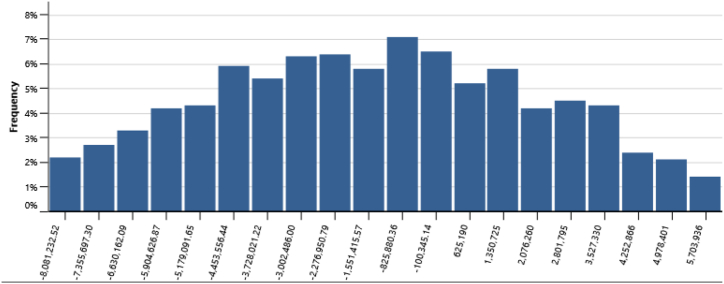
Distribution of RUPTL scenario – NPV.

**Fig. 18:**
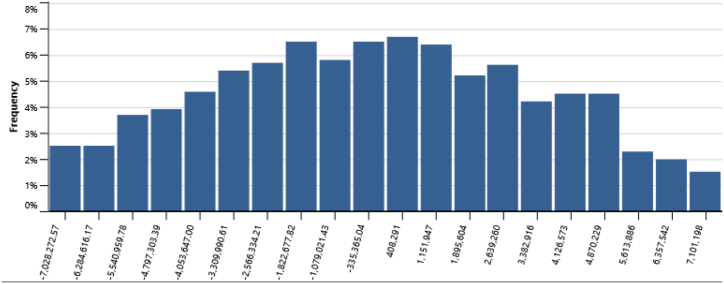
Distribution of proposed case scenario – NPV.

**Fig. 19:**
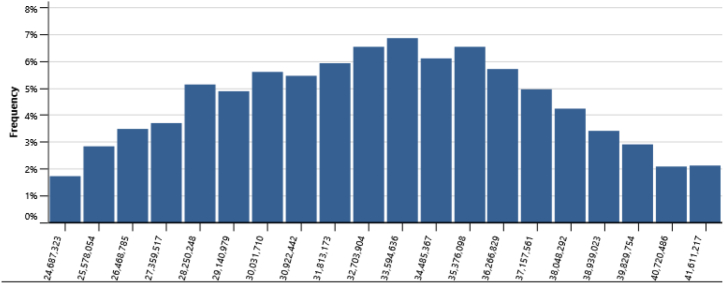
Distribution of cost-savings scenario -NPV.

**Fig. 20:**
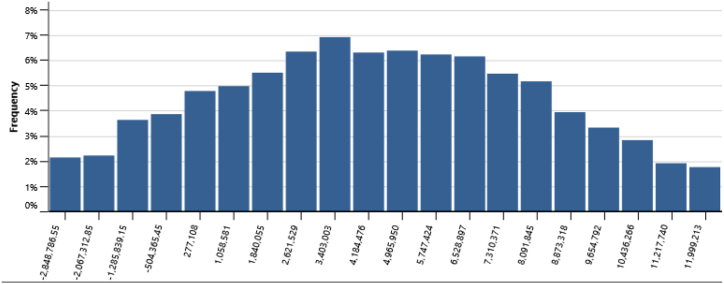
Distribution of clean energy scenario – NPV.

Table 14 presents the risk analysis results with Monte Carlo method utilizing RETScreen, collectively gathered from Fig. 16, Fig. 17, Fig. 18, Fig. 19, and Fig. 20. The table shows the probabilities of failure (NPV <0) and probabilities of success (NPV >0) for distinct project conditions. The base case, RUPTL, and proposed case scenarios exhibit probabilities of success at 30%, 41%, and 50%, respectively. Despite the three scenarios having probabilities of failure below or equal to 50%, it is noteworthy that the proposed case demonstrates a comparatively lower probability of failure when compared with RUPTL and the base case. It is also worth noting, that even after improvement, based on the IPPs perspective, the project is likely to result in 50% probabilities of success. This outcome signifies the project's lack of profitability in the absence of cost-savings and clean energy incentives. Consequently, strategic adjustments are imperative to align the project with financial viability and sustainability goals. Conversely, the clean energy scenario indicates that the introduction of a 10 USD/tCO_2_ incentive is pivotal in enhancing the project's success probabilities by 37%. Therefore, it is recommended to introduce clean energy incentives to make PV project financially viable for investors outside of PLN, in an attempt to accelerate Indonesia's energy transition. Based on the cost-savings results, it is recommended to revise PLN's RUPTL plan, specifically adjusting the initial capacity from 6 MW to 10 MW, with a subsequent expansion to 16 MW. This adjustment is anticipated to yield higher tariffs and, consequently, elevate the project's NPV. The cost savings scenario result underscores the project's potential for high profitability, as evidenced by a 100% probability of success derived from significant cost-savings.

**Table 14:** Risk analysis results for all scenarios.

Scenarios	Probabilities of Failure (NPV <0)	Probabilities of Success (NPV >0)
Base Case	70%	30%
RUPTL	59%	41%
Proposed Case	50%	50%
Cost Savings	0%	100%
Clean Energy	13%	87%

### 4.5 Discussion

Despite the improvement in the proposed case scenarios, from the IPPs perspective, the project is still considered unfeasible, as evidenced by its negative NPV value and an IRR value below the applied discount rate. This result is attributed to tariff limitation and lacks of incentive, hindering the project's profitability. Furthermore, the tariff employed in this study is the ceiling tariff or maximum tariff, introducing the possibility that the realized Power Purchase Agreement (PPA) may be lower than the tariff used in this study. Consequently resulting in a more negative NPV value. The inadequacy is also reflected in the country's solar power plant utilization, falling short behind its target due to low involvement of investors. Furthermore, when comparing the new tariffs [10] to the previous tariff, it is found that the new renewable energy tariffs are considerably lower compared to the previous tariffs [67]. The previous tariffs were set at 85% of the region's production cost, which, in the case of Nias, amounted to approximately 0.16 USD/kWh, significantly higher compared to the new tariffs. Additionally, our findings reveal that Indonesia's PV tariffs are relatively lower in comparison with other countries such as Iran and China [30,38]. Under the new renewable energy tariffs, the tariff for the 26 MW PV power plant is only around 0.0595 USD/kWh, making Indonesia's renewable energy sector less attractive to investors. In order to achieve a 23% renewable energy mix by 2025, it is crucial to establish more competitive renewable energy tariffs in Indonesia's energy sector. To address this issue, a clean-energy incentive is needed as a strategic approach to incentivize investors. The clean-energy scenario results proves that an introduction of 10 USD/tCO_2_ clean-energy incentives would increase the project's NPV to 4,393,516, significantly improved the project's feasibility. One example that could be implemented by Indonesian government is emissions incentives, which proven to be effective to lower GHG emissions [1,68,69]. Emission incentives such as emission trading system works on the “cap and trade” principle, where the overall volume of particular greenhouse gases that can be emitted by power plants, factories and aviation sector is limited by a “cap” on the number of emission allowance [70]. Power plants, factories and aviation sectors purchase or receive emissions allowances within the cap, which they can exchange with one another as necessary [71]. This incentive will bring more competitiveness in Indonesia's energy market, since not only the low-emissions that will be rewarded but there is also penalty for excess emission which brings more competitiveness into Indonesia's energy market. It is worth mentioning, that the incentives for GHG emissions is aligned with Indonesia's vision in the Conference of Parties of UNFCCC-United Nations of Framework Convention on Climate Change to decrease 29% GHG emissions in 2030 [72]. The new renewable energy regulation stated that the tariff will be evaluated yearly, which the authors hope that this study can contribute to the evaluation.

Conversely, based on cost-savings scenario, reflecting the PLN's perspective, this project is considered very profitable due to the significant benefits resulted from the de-dieselization, a factor contributing to the high cost of electricity production [66]. The cost-savings scenario's equity payback period, internal rate of return, and cost-savings are relatively similar to the findings of Pan's study [30]. However, the NPV results of this study are relatively high compared to Pan's due to the difference in the discount rate employed. In this research, a discount rate of 7% was used, whereas Pan employed a 12% discount rate, which ultimately resulted in a relatively higher NPV. It is also worth noting that the initial cost of PV power plants in China is relatively lower compared to this study due to the different prices of electrical components such as PV panels and inverters. Clean-energy scenario results proved that an emission reduction incentive is needed to make the project financially feasible for IPPs. This result is aligned with [34,38,65], proving that emission reduction incentives have a key role in improving PV project financial viability. When comparing this research to Kassem's [24], there is a relatively disparate results in terms of initial cost and LCOE due to the difference in conditions. For example, Nias is not a densely populated island where land prices are relatively cheap compared to Cyprus. In addition, the LCOE result of this study exhibits a comparatively higher value when compared to Rehman's study [26] due to solar irradiance differences which resulted in a difference in capacity factor and electricity output. Furthermore, the absence of renewable energy incentives that cover the initial cost has also contributed to the LCOE difference between this study and Rehman's study. However, it is crucial to highlight that the initial cost of PV power plants has experiences a substantial reduction over the past decade. A summary of results in comparison to other studies are summarized in Table 15.

**Table 15:** Results comparison with previous studies.

Author	Year	Scenario	Research Scope		Tariffs and Incentives			Costs			Research Results			
			Region	PV Capacity (MW)	Cost Savings (USD/kWh)	RE Incentives	Electricity Tariffs (USD/kWh)	Initial Costs (USD/kW)	O&M Costs (MUSD per annum)	NPV (MUSD)	Simple Payback Period (Years)	Equity Payback Period (Years)	Internal Rate of Return (%)	LCOE (USD/kWh)
This research	2023	Base Case	Indonesia	26			0.0570	999.03	0.338	−1.460	10.4	17.8	5.6	0.0595
	2023	RUPTL	Indonesia	26			0.0573	999.03	0.338	−1.262	10.4	17.6	5.8	0.0595
	2023	Proposed Case	Indonesia	26			0.0594	999.03	0.338	−0.062	10	16.7	6.9	0.0595
	2023	Cost Savings	Indonesia	26	0.0572		0.0594	999.03	0.338	33.002	4.8	2.2	41.8	0.0595
	2023	Clean Energy	Indonesia	26		10 USD/tCO_2_	0.0594	999.03	0.338	4.393	8.7	10.7	11.3	0.0595
[48]	2014	Makassar	Indonesia	15.81		0.25 USD/kWh Feed in Tariff		4600	0.050			16.7	10.0	
[26]	2017	Bisha Region	Saudi Arabia	10		30–70% from initial cost		1304	0.500	5.297		17.6	9.8	0.0183
[24]	2020	Lefkosa Region	Northern Cyprus	100				2700		52.338	17	7.8	16.3	0.0933
[38]	2012	First Scenario	Iran	0.008			0.0375	13,750	0.002		40.7	12.1	10.2	
	2012	Second Scenario	Iran	0.008			0.1750	13,750	0.002		16.9	8.0	18.0	
	2012	Third Scenario	Iran	0.008		20% incentives from initial cost 30 USD/tCO_2_	0.1750	13,750	0.002		12.3	6.0	21.9	
[30] a	2017	Fixed-tilt	China	200	0.078	5.81 USD/tCO_2_	0.097/kWh	397.83	0.616	127.240	3.5	1.6	65	
	2017	Single-axis	China	200	0.078	5.81 USD/tCO_2_	0.097/kWh	493.88	0.616	139.614	3.7	1.7	58	
	2017	Double-axis	China	200	0.078	5.81 USD/tCO_2_	0.097/kWh	589.93	0.616	125.930	4.3	2.2	50	

a Converted from CNY to USD – 1 USD = 7 CNY.

## 5 Conclusion

Based on the findings, it is recommended that the initial plan of PLN to construct a 6 MW initial and 20 MW expansion PV power plant (RUPTL scenario) be revised to a 10 MW initial and 20 MW expansion PV power plant (proposed case scenario). The proposed case scenario demonstrates greater cash flows, higher NPV, and a shorter equity payback period, resulting in a more profitable project. The projected LCOE of the power plant is 0.0595 USD/kWh, indicating a significant potential reduction in the average electricity production costs in Nias. Recent increases in fuel prices have significantly raised the production costs of diesel power plants, with an average cost of 0.396 USD/kWh in 2021. Based on those considerations, PV power plant appears to be a cost-effective solution for electricity generation in remote region, as most of remote regions are primarily reliant on diesel power plants. Consequently, the integration of PV power plant in Nias is considered very profitable from PLN perspective, due to significant cost-savings derived from de-dieselization process, as shown in the cost-savings scenario results. Considering circumstances mentioned earlier, this study recommends PLN to accelerate the de-dieselization program. This initiative will lead to not only the attainment of environmental sustainability but also in the reduction of Indonesia's energy production cost, thereby making energy more accessible for everyone.

However, from IPPs perspective, the new PV tariff alone is inadequate to make the project financially feasible due to tariffs limitation and the absence of incentives. While PLN might benefit from the cost-savings of the de-dieselization process, it does not confer financial benefits to IPPs. Limitation of tariff and the lacks of incentive may decelerate the de-dieselization process due to challenges associated with the project's profitability from IPPs perspective. To address this issue, our study propose a clean energy incentive. Our analysis showed that a 10 USD/tCO_2_ is sufficient to make the project profitable based on IPP perspective, increasing the project NPV from −61,714 USD to 4,393,516 USD. Furthermore, according to the risk analysis, the introduction of clean energy incentive would increase the probability of success from 50% to 87%. Therefore, this study recommends the introduction of clean energy incentive to attract more investors in Indonesia's PV power plant sector.

There are several suggestions for further research to provide more insights. The author suggests further research to incorporate climate analysis using field data, as it will provide a more accurate estimation of the energy produced by the PV power plant and a more realistic assessment of environmental conditions. A more technically oriented approach is also suggested, such as including an analysis of the decline in the efficiency of PV panels over their operational lifespan, to measure the reliability and the true cost of PV power plants. The author also suggests studies in different locations and with other renewable energy technologies, using the newly implemented renewable energy tariffs, to gain more insights about the adequacy of these tariffs in other locations and other technologies. Nevertheless, one can expect the findings of this study to be applicable in other locations as well, emphasizing the importance of specific renewable energy technologies in reducing electricity production costs hence improving energy accessibility, which will hopefully contribute in achieving Indonesia's energy transition.

## Funding statement

This research did not receive any specific grant from funding agencies in the public, commercial, or not-for-profit sectors.

## Data availability statement

Data included in article/supp. material/referenced in article.

## CRediT authorship contribution statement

**Hendry Timotiyas Paradongan:** Writing – review & editing, Writing – original draft, Methodology, Formal analysis, Data curation, Conceptualization. **Dzikri Firmansyah Hakam:** Validation, Supervision, Methodology, Formal analysis, Conceptualization. **Sudarso Kaderi Wiryono:** Validation, Supervision, Conceptualization. **Iswan Prahastono:** Supervision, Resources, Conceptualization. **Indra A. Aditya:** Supervision, Resources, Project administration, Formal analysis, Conceptualization. **Kevin M. Banjarnahor:** Validation, Supervision, Investigation, Formal analysis, Conceptualization. **Ngapuli Irmea Sinisuka:** Validation, Supervision, Methodology, Formal analysis, Conceptualization. **Ayodele Asekomeh:** Validation, Conceptualization.

## Declaration of competing interest

The authors declare that they have no known competing financial interests or personal relationships that could have appeared to influence the work reported in this paper.

## References

[1] IEA (2022). https://www.iea.org/reports/world-energy-outlook-2022.

[2] ADB (2020). “Energy sector assessment, strategy, and road map: Indonesia,” Manila.

[3] IEA (2020). https://www.iea.org/countries/indonesia.

[4] Coordinating Ministry of Economic Affairs (2023). Pemerintah Terus Mendorong Percepatan Transisi Energi di Dalam Negeri Guna Mencapai target net zero emission pada 2060. https://www.ekon.go.id/publikasi/detail/4996/pemerintah-terus-mendorong-percepatan-transisi-energi-di-dalam-negeri-guna-mencapai-target-net-zero-emission-pada-2060.

[5] ESDM (2021). Pemerintah Optimis EBT 23% Tahun 2025 Tercapai. https://www.esdm.go.id/id/berita-unit/direktorat-jenderal-ketenagalistrikan/pemerintah-optimistis-ebt-23-tahun-2025-tercapai.

[6] Govindarajan L., Bin Mohideen Batcha M.F., Bin Abdullah M.K. (2023). Solar energy policies in southeast Asia towards low carbon emission: a review. Heliyon.

[7] Kanugrahan S.P., Hakam D.F. (2023). Long-term scenarios of Indonesia power sector to achieve Nationally determined contribution (NDC) 2060. Energies.

[8] Hakam D.F., Nugraha H., Wicaksono A., Rahadi R.A., Kanugrahan S.P. (2022). Mega conversion from LPG to induction stove to achieve Indonesia's clean energy transition. Energy Strateg. Rev..

[9] ESDM (2022). https://www.esdm.go.id/en/media-center/news-archives/renewables-power-plants-cod-within-target-says-official.

[10] President of Indonesia (2022). https://drive.esdm.go.id/wl/?id=o8WDm5f2AXpP9Awt2y4CFnvB3t2JdOAf.

[11] Makkiabadi M. (2021). Performance evaluation of solar power plants: a review and a case study. Processes.

[12] Hirbodi K., Enjavi-Arsanjani M., Yaghoubi M. (2020). Techno-economic assessment and environmental impact of concentrating solar power plants in Iran. Renew. Sustain. Energy Rev..

[13] Nili M., Seyedhosseini S.M., Jabalameli M.S., Dehghani E. (2021). A multi-objective optimization model to sustainable closed-loop solar photovoltaic supply chain network design: a case study in Iran. Renew. Sustain. Energy Rev..

[14] Al Garni H.Z., Awasthi A. (2017). Solar PV power plant site selection using a GIS-AHP based approach with application in Saudi Arabia. Appl. Energy.

[15] Wyban C.A. (2020).

[16] Makkiabadi M., Hosseinzadeh S., Nezhad M.M., Sohani A., Groppi D. (2021). Techno‐economic study of a new hybrid solar desalination system for producing fresh water in a hot–arid climate. Sustain. Times.

[17] Ali T., Ma H., Nahian A.J. (2019). An analysis of the renewable energy technology selection in the southern region of Bangladesh using a hybrid multi-criteria decision making (MCDM) method. Int. J. Renew. Energy Res..

[18] Reddy K.S., Veershetty G. (2013). Viability analysis of solar parabolic dish stand-alone power plant for Indian conditions. Appl. Energy.

[19] ESDM (2012). Sun for photovoltaic power plant in Indonesia. Ministry of Energy and Mineral Resources of Indonesia.

[20] ESDM (2020).

[21] Kanugrahan S.P., Hakam D.F., Nugraha H. (2022). Techno-economic analysis of Indonesia power generation expansion to achieve economic sustainability and net zero carbon 2050. Sustain. Times.

[22] PLN (2021). https://web.pln.co.id/statics/uploads/2023/02/SR-PLN-2021-Low-revisi-0111.pdf.

[23] PLN (2021). “Statistik PLN 2021,” Jakarta. https://web.pln.co.id/stakeholder/laporan-statistik.

[24] Kassem Y., Çamur H., Alhuoti S.M.A. (2020). Solar energy technology for northern Cyprus: assessment, statistical analysis, and feasibility study. Energies.

[25] Kelly E., Medjo Nouadje B.A., Tonsie Djiela R.H., Kapen P.T., Tchuen G., Tchinda R. (2023). Off grid PV/Diesel/Wind/Batteries energy system options for the electrification of isolated regions of Chad. Heliyon.

[26] Rehman S., Ahmed M.A., Mohamed M.H., Al-Sulaiman F.A. (2017). Feasibility study of the grid connected 10 MW installed capacity PV power plants in Saudi Arabia. Renew. Sustain. Energy Rev..

[27] IESR (2019). https://iesr.or.id/wp-content/uploads/2020/01/LCOE-Full-Report-ENG.pdf.

[28] (2021). Lazard. Lazard’s Levelized Cost of Energy v15.0.

[29] NREL (2020). https://maps.nrel.gov/slope/data-viewer?layer=lcoe.levelized-cost-of-electricity&res=county&year=2020&filters=%5B%5D.

[30] Pan Y., Liu L., Zhu T., Zhang T., Zhang J. (2017). Feasibility analysis on distributed energy system of Chongming County based on RETScreen software. Energy.

[31] Baccay Sy J., Haile A., Degife W. (2020). Feasibility study of a 100MW photovoltaic power plant at Bati, Ethiopia using RETScreen. Int. J. Sci. Res. Publ..

[32] Mondal A.H., Islam S. (2011). Potential and viability of grid-connected solar PV system in Bangladesh. Renew. Energy.

[33] Khalid A., Junaidi H. (2013). Study of economic viability of photovoltaic electric power for Quetta - Pakistan. Renew. Energy.

[34] Owolabi A.B., Nsafon B.E.K., Huh J.S. (2019). Validating the techno-economic and environmental sustainability of solar PV technology in Nigeria using RETScreen Experts to assess its viability. Sustain. Energy Technol. Assessments.

[35] Weida S., Kumar S., Madlener R. (2016). Financial viability of grid-connected solar PV and wind power systems in Germany. Energy Proc..

[36] Sreenath S., Sudhakar K., Af Y. (2021). 7E analysis of a conceptual utility-scale land-based solar photovoltaic power plant. Energy.

[37] EL-Shimy M. (2009). Viability analysis of PV power plants in Egypt. Renew. Energy.

[38] Mirzahosseini A.H., Taheri T. (2012). Environmental, technical and financial feasibility study of solar power plants by RETScreen, according to the targeting of energy subsidies in Iran. Renew. Sustain. Energy Rev..

[39] Rashwan S.S., Shaaban A.M., Al-Suliman F. (2017). A comparative study of a small-scale solar PV power plant in Saudi Arabia. Renew. Sustain. Energy Rev..

[40] Zandi M. (2017). Evaluation and comparison of economic policies to increase distributed generation capacity in the Iranian household consumption sector using photovoltaic systems and RETScreen software. Renew. Energy.

[41] Chowdhury N., Hossain C.A., Longo M., Yaïci W. (2020). Feasibility and cost analysis of photovoltaic-biomass hybrid energy system in off-grid areas of Bangladesh. Sustain. Times.

[42] Liu G., Rasul M.G., Amanullah M.T.O., Khan M.M.K. (2011). Feasibility study of stand-alone PV-wind-biomass hybrid energy system in Australia. Asia-Pacific Power Energy Eng. Conf. APPEEC.

[43] Mehmood A., Shaikh F.A., Waqas A. (2014). Modeling of the solar photovoltaic systems to fulfill the energy demand of the domestic sector of Pakistan using RETSCREEN software. Proc. 2014 Int. Conf. Util. Exhib. Green Energy Sustain. Dev. ICUE.

[44] Zhang X., Li M., Ge Y., Li G. (2016). Techno-economic feasibility analysis of solar photovoltaic power generation for buildings. Appl. Therm. Eng..

[45] Mondal M.A.H., Islam A.K.M.S. (2009). Techno-economic feasibility of grid connected solar PV system in Bangladesh. Proc. 1st Int. Conf. Dev. Renew. Energy Technol. ICDRET.

[46] Lee K.H., Lee D.W., Baek N.C., Kwon H.M., Lee C.J. (2012). Preliminary determination of optimal size for renewable energy resources in buildings using RETScreen. Energy.

[47] Sanni S.H., Mohammed K. (2018). Residential solar photovoltaic system Vs grid supply: an economic analysis using RETScreen^TM^. J. Sol. Energy Res..

[48] Fathoni A.M., Utama N.A., Kristianto M.A. (2014). A technical and economic potential of solar energy application with feed-in tariff Policy in Indonesia. Procedia Environ. Sci..

[49] Kassem Y., Camur H., Abughinda O.A.M. (2020). Solar energy potential and feasibility study of a 10MW grid-connected solar plant in Libya. Eng. Technol. Appl. Sci. Res..

[50] Al-Turjman F., Qadir Z., Abujubbeh M., Batunlu C. (2020). Feasibility analysis of solar photovoltaic-wind hybrid energy system for household applications. Comput. Electr. Eng..

[51] Tarigan Elisier (2018). Simulation and feasibility studies of Rooftop PV system for University Campus buildings in Surabaya, Indonesia. Int. J. Renew. Energy Res..

[52] Tarigan E., Djuwari, Kartikasari F.D. (2015). Techno-economic simulation of a grid-connected PV system design as specifically applied to Residential in Surabaya, Indonesia. Energy Proc..

[53] World Bank (2022). https://www.worldbank.org/en/country/indonesia/overview.

[54] ESDM (2022). *Ministry of Energy and Mineral Resources of Indonesia*. Ministry of Energy and Mineral Resources of Indonesia, Jakarta, Indonesia.

[55] ADB (2020).

[56] Bank Indonesia (2022). https://www.bi.go.id/id/statistik/informasi-kurs/jisdor/default.aspx.

[57] Syafrianto D., Banjar-Nahor K.M., Nugraha H., Hakam D.F., Hadi P.O., Hariyanto N. (2021). 2021 3rd International Conference on High Voltage Engineering and Power Systems (ICHVEPS).

[58] Miller A., Lumby B. (2012).

[59] Natural Resources Canada (2024).

[60] Asamoah S.S., Parbey J., Yankey I.K., Awuah A. (2023). Techno-economic assessment of a central grid-connected wind farm in Ghana using RETScreen® Expert. Heliyon.

[61] Bank Indonesia, “Data Inflasi.”.https://www.bi.go.id/id/statistik/indikator/data-inflasi.aspx (accessed January. 28, 2023)..

[62] Jasa Keuangan Otoritas (2023). https://ojk.go.id/id/kanal/perbankan/pages/suku-bunga-dasar.aspx.

[63] PLN (2021). Rencana Usaha Penyediaan Tenaga Listrik (RUPTL) PT PLN (Persero) 2021-2030. https://web.pln.co.id/statics/uploads/2021/10/ruptl-2021-2030.pdf.

[64] Rubinstein R.Y., Kroese D.P. (2016).

[65] Prahastono I., Paradongan H.T., Aditya I.A., Banjarnahor K.M., Sinisuka N.I. (2023). Photovoltaic power plant feasibility study based on Indonesia's renewable energy tariffs: a study of Nusa Penida solar power plant. Int. J. Energy Econ. Policy.

[66] Hakam D.F., Arif L., Fahrudin T. (2012). 2012 International Conference on Power Engineering and Renewable Energy (ICPERE).

[67] ESDM (2020). https://jdih.esdm.go.id/index.php/web/result/2032/detail.

[68] NCEE (2001).

[69] OECD (2004). Policies to reduce greenhouse gas emissions in Industry: Successful Approaches and Lessons Learned: Workshop report. OECD Pap..

[70] European Commisions (2021). Emissions cap and allowances. https://climate.ec.europa.eu/eu-action/eu-emissions-trading-system-eu-ets/emissions-cap-and-allowances_en.

[71] European Commisions (2023). EU emissions trading system (EU ETS). https://climate.ec.europa.eu/eu-action/eu-emissions-trading-system-eu-ets_en.

[72] ESDM (2019). https://jdih.esdm.go.id/storage/document/Kepmen-esdm-143-Thn2019RUKN2019.pdf.

